# Techniques for Analysis of Plant Phenolic Compounds

**DOI:** 10.3390/molecules18022328

**Published:** 2013-02-19

**Authors:** Ali Khoddami, Meredith A. Wilkes, Thomas H. Roberts

**Affiliations:** Department of Plant and Food Sciences, University of Sydney, Sydney, NSW 2006, Australia; E-Mails: ali.khoddami@sydney.edu.au (A.K.); meredith.wilkes@sydney.edu.au (M.A.W.)

**Keywords:** food analysis, phenolic compound, phenolic extraction technique, phenolic quantification method, HPLC, GC

## Abstract

Phenolic compounds are well-known phytochemicals found in all plants. They consist of simple phenols, benzoic and cinnamic acid, coumarins, tannins, lignins, lignans and flavonoids. Substantial developments in research focused on the extraction, identification and quantification of phenolic compounds as medicinal and/or dietary molecules have occurred over the last 25 years. Organic solvent extraction is the main method used to extract phenolics. Chemical procedures are used to detect the presence of total phenolics, while spectrophotometric and chromatographic techniques are utilized to identify and quantify individual phenolic compounds. This review addresses the application of different methodologies utilized in the analysis of phenolic compounds in plant-based products, including recent technical developments in the quantification of phenolics.

## 1. Introduction

Plant foods are rich sources of phenolics, which are molecules that can act as antioxidants to prevent heart disease [1,2,3], reduce inflammation [4,5,6], lower the incidence of cancers [7,8,9,10] and diabetes [11,12], as well as reduce rates of mutagenesis in human cells [7,13,14]. The protection afforded by the consumption of plant products such as fruits, vegetables and legumes is mostly associated with the presence of phenolic compounds. 

**Figure 1:**
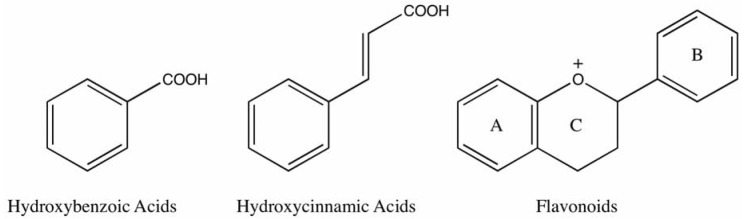
Basic structures of phenolic acids and flavonoids.

Phenolic compounds are synthesized in plants partly as a response to ecological and physiological pressures such as pathogen and insect attack, UV radiation and wounding [15,16,17,18]. The basic structural feature of phenolic compounds is an aromatic ring bearing one or more hydroxyl groups (Figure 1) [19]. Plant phenolic compounds are classified as simple phenols or polyphenols based on the number of phenol units in the molecule. Thus, plant phenolics comprise simple phenols, coumarins, lignins, lignans, condensed and hydrolysable tannins, phenolic acids and flavonoids [20].

Flavonoids are some of the most common phenolics, widely distributed in plant tissues, and often responsible alongside the carotenoids and chlorophylls for their blue, purple, yellow, orange and red colors. The flavonoid family includes flavones, flavonols, iso-flavonols, anthocyanins, anthocyanidins, proanthocyanidins and catechins [21,22]. All flavonoids are derived from the aromatic amino acids, phenyalanine and tyrosine, and have three-ringed structures [23]. Variation in flavonoid structure arises from the scale and pattern of hydroxylation, prenylation, alkalinization and glycosylation reactions that alter the basic molecule [24].

Phenolic acids are one of the other main phenolic classes within the Plant Kingdom and occur in the form of esters, glycosides or amides, but rarely in free form. Variation in phenolic acids is in the number and location of hydroxyl groups on the aromatic ring [25]. Phenolic acids have two parent structures: hydroxycinnamic and hydroxybenzoic acid. Hydroxycinnamic acid derivatives include ferulic, caffeic, *p*-coumaric and sinapic acids, while hydroxybenzoic acid derivatives consist of gallic, vanillic, syringic and protocatechuic acids.

Another major class of phenolic compounds is the cell wall phenolics. They are insoluble and found in complexes with other types of cell components. The two main groups of cell wall phenolics are lignins and hydroxycinnamic acids [26,27]. These compounds play a critical role in the cell wall during plant growth by protecting against stresses such as infection, wounding and UV radiation [28]. Tannins can be divided into two groups, hydrolysable tannins and condensed tannins, and have great potential to form oxidative linkages to other plant molecules.

Several recent reviews are available on the characterization of phenolics in foods [1,22,23,24,28]. Here we review several techniques to extract and analyse plant phenolic compounds. The most important steps for the analysis of phenolic compounds are sample preparation and extraction, followed by classification and quantification using spectrophotometry, gas chromatography (GC), high performance liquid chromatography (HPLC) or capillary electrophoresis (CE) methods.

## 2. Sample Preparation

Plants foods (including fruits, cereal grains, legumes and vegetables) and beverages (including tea, coffee, fruit juices and cocoa) are major sources of phenolics in the human diet. The preparation and extraction of phenolic compounds from this wide range of samples depends mostly on the nature of the sample matrix and the chemical properties of the phenolics, including molecular structure, polarity, concentration, number of aromatic rings and hydroxyl groups. Variation in the chemistry of phenolics in a sample is related to the concentration of simple and complex polyphenolic compounds and the different proportions of phenolic acids, flavonoids, anthocyanins and proanthocyanins (among others). Thus, it is difficult to choose a single method of preparation and extraction for phenolics for many plant products.

Complexes with proteins, carbohydrates or other elements hinder complete extraction of some phenolics. For some preparation techniques, plant samples need to be dried using freeze-drying, air-drying or oven-drying. For example, Sejali and Anuar [29] indicated that higher amounts of phenolics can be extracted in shade air-dried neam leaf than from oven-dried samples. Dried samples are milled or ground to obtain a certain particle size, whereas liquid samples are treated by centrifugation, filtration and purification using a separation system when required. Higher extraction yields of phenolics are achieved by milling the sample into smaller particle sizes, thereby improving enzymatic action and extraction [30]. Defatting processes can be applied to remove oil from lipid-containing samples. For instance, Weidner *et al.* [31] defatted the ground seeds of grape to simplify phenolic extraction using hexane. In general, milling into small particle size (in combination with drying and de-fatting where appropriate) is advised for the most complete sample preparation prior to extraction.

## 3. Overview of Phenolic Extraction

Complete extraction of phenolic compounds is the next critical step after sample preparation. The most common techniques to extract phenolics employ solvents, either organic or inorganic. Several parameters may influence the yield of phenolics, including extraction time, temperature, solvent-to-sample ratio, the number of repeat extractions of the sample, as well as solvent type. Furthermore, the optimum recovery of phenolics is different from one sample to the other and relies on the type of plant and its active compounds. The choice of extraction solvents such as water, acetone, ethyl acetate, alcohols (methanol, ethanol and propanol) and their mixtures [32] will influence the yields of phenolics extracted. For instance, a high yield of phenolics can be extracted from sorghum leaf using water [33], while extraction of phenolics from wheat bran requires 80% aqueous ethanol [34]. In another example, an investigation into the effect of different solvents on extraction of phenolics from aerial parts of *Potentilla atrosanguinea* showed that 50% aqueous ethanol was more efficient than pure or 50% aqueous forms of methanol, and acetone [35]. In contrast, the highest levels of phenolics are extracted from *Vitis vinifera* wastes and sunflower meal using pure methanol and 80% aqueous acetone, respectively [36,37]. These differences could be due to the properties of the phenolic components of the plants concerned.

In addition to selecting the optimal extraction solvent, there are two other important parameters that affect the yield of phenolics extracted from plant foods: time and temperature. Normally, increasing time and temperature promote analyte solubility; however, plant phenolics are generally degraded or undergo undesirable reactions such as enzymatic oxidation by extended extraction times and high temperatures [38,39]. Naczk *et al.* [40] demonstrated that the optimum extraction time and temperature to extract phenolics from canola meal is 2 min (2 × 1 min) at room temperature. The solvent-to-sample ratio and the number of replicate extractions performed for each sample also affect the recovery of phenolics. Increasing the solvent-to-sample ratio promotes phenolic extraction from plant samples but determining the optimum ratio is advisable so that solvent input and saturation effects of solvent by the phenolics are minimized. Al-Farsi and Lee [41] reported that a 60:1 ratio of solvent to sample in a two-stage procedure is sufficient to extract most phenolics from plant tissues.

Sample matrix and particle size also strongly influence phenolic extraction from plant materials. Phenolics may bind to other sample elements such as carbohydrates and proteins [42]. These linkages can be hydrolyzed by addition of enzymes, thereby promoting the release of bound phenolics [42]. Acidic and alkaline hydrolysis are also employed in the isolation of phenolics from plants and plant products and are important for the stability of the phenolics in the extract [43,44]. Flavonoid aglycones have been identified by acidic hydrolysis of the glycosidic residues bound to the flavonoid nucleii in 20 plant sources [43]. In another study, Davidov-Pardo *et al.* [39] reported that a pH of 4–5 was associated with greater stability of catechins and their isomers than alkaline and more acidic conditions. General considerations/techniques for extraction of specific classes of phenolic compounds will be discussed in more detail in the following sections.

### 3.1. Phenolic Acid Extraction

Phenolic acids generally exist in a free, esterified or glycosylated form in plants. Ayumi *et al.* [45] extracted free phenolic acids in rice using 70% ethanol at room temperature followed by centrifugation. The extract was then treated with 4 M HCl to reduce the pH to 2–3 and the phenolic fraction separated using ethyl acetate and dried with anhydrous disodium sulfate. The bound or esterified phenolic acids of rice were extracted by removing the free phenolic acids and lipid using 70% ethanol and hexane, respectively. The dried ethyl acetate fractions were treated with 1 M NaOH containing 0.5% sodium borohydride (NaBH_4_) to liberate esterified phenolic acids in a stream of N_2_ gas, followed by centrifugation to obtain a clear supernatant. Degradation of phenolic acids during alkaline hydrolysis can be prevented by adding EDTA or ascorbic acid [46]. 

Free, esterified and glycosylated phenolic acids have been separated from wheat, rye and triticale [47]. Phenolic compounds were extracted using 80% methanol for 15 min at 80 °C and the extract concentrated by evaporating the organic solvent. An aqueous suspension of the extract was then prepared and adjusted to pH 2 with 6 M HCl and the free phenolic acids extracted using diethyl ether. The residue of the suspended extract was neutralized and dissolved in 20 mL of NaOH (2 M) for 4 h under N_2_. After alkaline hydrolysis, the extract was acidified again to pH 2. Esterified phenolic acids, derivatized by mixing the extract with diethyl ether, were isolated using a separating funnel. To release phenolic acids from glycosylated forms, 15 mL of 6 M HCl was added to the remaining aqueous fraction and the mixture kept in 100 °C for 1 h under N_2_. Finally, the released phenolic acids were isolated using diethyl ether. Apart from ethanol, mixtures of water with methanol, acetone and chloroform may be used for phenolic acid extraction from plant-based products [24]. These studies show that free and bound forms of phenolic acids can be extracted sequentially from plant samples.

### 3.2. Flavonoid Extraction

Flavonoids are highly bioactive compounds found in both edible and non-edible plants. They are often extracted with methanol, ethanol, acetone, water or mixtures of these solvents using heated reflux extraction methods [23,48,49,50]. Following extraction, the flavonoid glycosides are frequently hydrolyzed into the aglycone forms by applying HCl under N_2_. Haghi and Hatami [43] extracted flavonoids from different types of herbal plant materials with 50% methanol acidified by 1.2 M HCl. Ascorbic acid was added to prevent oxidation of the mixture. The hydrolysis of the flavonoid glycosides was carried out for 2 h at 80 °C [43]. Tsimogiannis *et al.* [51] extracted flavonoids from dried and defatted plant material in diethyl ether and filtered pooled samples for HPLC analysis. Flavonoids can also be extracted from plant material with 62.5% methanol. The extract is acidified with 6 M HCl under N_2_ at 90 °C for 2 h to obtain flavonoid glycones [52]. Wu *et al.* [53] focused on optimization of enzymatic extraction of flavonoids from celery stalks. The pulpy aqueous homogenate was mixed with 1 N HCl or NaOH to adjust the pH and the mixture incubated at the desired temperature. A complex mixture of enzymes was added to the sample under stirring at 150 rpm. The enzymes were then inactivated by heating at 90 °C for 10 min and the supernatant of the centrifuged mixture was collected for total flavonoid determination.

Biesaga [50] extracted flavonoids in maize samples using heated reflux, microwave-assisted extraction (MAE), ultrasonic-assisted extraction (UAE) and maceration and compared the stability of the extracted compounds. The highest stability of the extracted flavonoids in methanol-water (60:40 v/v) was for compounds extracted with traditional heated reflux in a water bath and MAE within 1 min under 160 W.

### 3.3. Anthocyanin/Proanthocyanidin Extraction

Anthocyanins are the most common pigments in nature and can be extracted with acidified solvents like water, acetone, ethanol, methanol or mixtures of aqueous solvents [54,55,56,57]. The acid in the solvents acts to rupture cell membranes and release anthocyanins; however, this harsh chemical treatment may break down the innate anthocyanin structure. It is therefore important to acidify solvents with organic acids (formic or acetic acid) rather than mineral acids such as 0.1% HCl [58]. Bridgers *et al.* [59] reported that extraction of anthocyanin from purple-fleshed sweet potato was more effective with acidified methanol and ethanol than non-acidified solvents. According to Awika *et al.* [56], the extraction of anthocyanin from black sorghum with acidified methanol was significantly higher than with aqueous acetone. This result is in agreement with Lee *et al.* [60], who used the same solvents to extract anthocyanin from three American *Vaccinium* species. 

Methanol is indeed the most common and effective solvent for extracting anthocyanins; however, it is an environmental pollutant and more toxic than other alcohols [59,61]. Thus ethanol is preferred for the recovery of anthocyanins from plant material to use as natural colorants or nutraceuticals [62]. Apart from acidified solvent extraction, sulfur water (aqueous SO_2_) has also been used to extract anthocyanins from plant materials such as red grape skin and black currants [63,64].

Proanthocyanidins are a group of polymerized polyphenols commonly referred to as condensed tannins [65]. They are found naturally in grape seed and skin, malt, apple juice, cider, mangosteen pericarp, hops, berries, pine bark, chocolate, sorghum and sea bark [65,66]. For proanthocyanidin extraction, organic solvents are usually used, including ethanol as well as methanol and acetone [67,68]. For example, Hernández-Jiménez *et al.* [67] reported that ethanol is the best solvent for proanthocyanidin extraction from grape seed. Ionic liquids (molten salts), which are chemically stable, easily recycled and non-flammable, are a new alternative solvent for extracting proanthocyanidins. They have been used to extract proanthocyanidins from *Larix gmelini* bark using microwave-assisted extraction methods, resulting in higher yields of proanthocyanidin when compared to conventional extraction methods with organic solvents [69].

## 4. Modern Extraction Techniques for Phenolics

Sample preparation and removal of unwanted substances for accurate quantification of phenolics is important, but the extraction procedure is the primary determinant for the separation and recovery of phenolics. As mentioned earlier, extraction is generally influenced by the sample nature, particle size, solvent type as well as extraction techniques employed.

Soxhlet, heated reflux extraction and maceration are conventional procedures frequently used to recover phenolics from solid samples. The Soxhlet and heated reflux methods are normally performed at 90 °C for several hours while maceration is performed over days at ambient temperature. These methods are simple, require relatively cheap apparatus and result in adequately high phenolic extraction rates [35,50,70]. Castro-Vargasa *et al.* [70] reported that the highest total phenolic content of Guava seed extract was achieved using Soxhlet extraction techniques. In another study, phenolic compounds from seeds of three wild grapevines were successfully extracted using the Soxhlet technique [31]. While there are many positive aspects of this method, there are substantial disadvantages, including: (1) the need to use large volumes of hazardous organic solvents, which are environmental pollutants and health hazards; (2) long extraction times and (3) interference with, and degradation of, targeted components due to both internal and external factors such as light, air, high temperatures and enzymatic reactions [71,72,73].

Soxetec is a modified Soxhlet extraction method. The advantages of this technique over normal Soxhlet or heated reflux systems are a low consumption of organic solvents and shorter extraction times, as well as the ability to recycle solvents [74]. Maceration has the same disadvantages as other conventional extraction methods, and is characterized by low efficiency of phenolic extractions [35,75]. Due to problems associated with conventional extraction procedures, a demand for alternative techniques for extraction of phenolic compounds has arisen. The use of ultrasound-assisted extraction (UAE), microwave-assisted extraction (MAE), ultrasound-microwave-assisted extraction (UMAE), supercritical fluid extraction (SFE), sub-critical water extraction (SCWE) and high hydrostatic pressure processing (HHPP) is increasing. These methods shorten extraction times, decrease the release of toxic pollutants through reducing organic solvent consumption, and are relatively simple to perform.

### 4.1. Ultrasound-Assisted Extraction (UAE)

Ultrasonic radiation, which has frequencies higher than 20 kHz, facilitates the extraction of organic and inorganic compounds from solid matrices using liquid solvents. Sonication is the production of sound waves that create cavitation bubbles near the sample tissue, which break down to disrupt cell walls, thereby releasing cell contents [76,77]. An appropriate solvent is mixed with a sample and sonicated under controlled temperature for a specified time. Extract recovery is influenced not only by sonication time, temperature and solvent selection, but also by wave frequency and ultrasonic wave distribution [78]. Ultrasound has been used in both static and dynamic modes to extract phenolics from plant materials [79]. A static system is a closed-vessel extraction for which no continuous transfer of solvent occurs. In dynamic extraction, fresh solvent is supplied continuously, which allows efficient adsorption of analytes and their effective transfer from the extraction vessel. Continuous transfer of extracted analytes prevents degradation of any thermo-labile compounds by the heat associated with sonication [80,81,82].

Probe and bath systems are the two most common ways of applying ultrasound waves to the sample. Probe sonicators are constantly in contact with the sample and make reproducibility and repeatability difficult. In addition, the risk of sample contamination and foam production is higher. Bath sonicators can act on a range of samples simultaneously and allow for higher reproducibility [83].

Compared to conventional methods, UAE is one of the most simple, inexpensive extraction systems and can be operated rapidly in a broad range of solvents for large-scale preparations suited for industrial purposes [84]. As a method to extract phenolic compounds from *Potentilla atrosanguinea* and *Pinus radiata*, UAE has been shown to be more effective than maceration but less effective than heated reflux, MAE and UMAE methods [35]. Many studies have involved extraction of biologically active compounds from different types of samples using these techniques (Table 1).

**Table 1:** Extraction of biologically active compounds using UAE.

Sample	Solvent	Extraction time (min)	Phenolic class	Yield (mg GAE ^b^/g)	Reference
*Puerariae lobatae* radix	Ethanol 80%	55	Isoflavones	128	[53]
*Vitis vinifera*	Methanol	60	TPC ^a^ and flavonoid	55.90	[36]
*Galla chinensis*	Ethanol 70%	40	Tannin	491.2	[85]
Sunflower meal	Acetone 80%	30	TPC ^a^	30.93	[37]
Orange peel	Ethanol 80%	30	TPC ^a^	2.758	[86]
Satsuma mandarin peel	Methanol 80%	60	Hesperidine	1.446	[87]
Aerial parts of *Potentilla atrosanguinea*	Ethanol 50%	60	TPC ^a^	27.80	[35]
Soy beans	Ethanol 40–60%	20	Isoflavones	1.353	[88]

^a^ Total phenolic content; ^b^ Gallic acid equivalent.

### 4.2. Microwave-Assisted Extraction (MAE)

Microwaves have been applied widely in research on secondary plant metabolites for decades [89]. Microwaves are non-ionizing radiation with wavelengths ranging from as long as one meter to as short as one millimeter (frequencies between 300 MHz and 300 GHz). Microwaves induce molecular motion in materials or solvents with dipoles, resulting in sample heating [90]. The heating causes plant cells to lose moisture through evaporation; the steam generated swells and eventually ruptures the cells, releasing their active components [78]. Apart from dipole materials of the plant cell, such as water molecules, the dipole rotation of the solvent molecules under the rapid change of electric field plays an important role in MAE. During radiation, the wave electronic module changes 4.9 × 10^4^ times/s and the solvent molecules are induced to align themselves in the normal phase with the electric field. At such a great change in the speed of the electric phase the solvent molecules fail to realign and begin to vibrate, heating the sample due to frictional forces [91].

The advantages of MAE techniques compared to conventional methods (such as maceration and heat reflux) include reduced use of organic solvents, reduced extraction time (generally less than 30 min) and increased extraction yields [92].

The hot solvents generated in MAE penetrate easily into the matrix and extract compounds from the lysed plant cells. For thermolabile samples, transparent solvents such as hexane, chloroform and toluene, or mixtures with non-transparent solvents, prevent degradation. It is important to select suitable solvents based on their boiling points, dissipation and dielectric properties [93]. The most commonly applied solvents in MAE are presented in Table 2. Polar solvents have a higher dielectric constant than non-polar solvents and can absorb more microwave energy, which can result in a higher yield of phenolics. 

**Table 2:** Important properties of some solvents commonly used in MAE.

Solvent	Formula	Boiling point (°C)	Dielectric constant ^a^	Dissipation factor
Acetonitrile	C_2_H_3_N	81.60	37.50	620
Water	H_2_O	100	78.30	1570
Ethanol	C_4_H_8_O_2_	78.5	24.30	2500
Acetone	C_3_H_6_O	56.2	20.70	5555
Methanol	CH_4_O	64.6	32.60	6400
2-Propanol	C_4_H_8_O	98	19.90	6700

^a^ Determined at 20 °C [94,95].

The dissipation factor is also important to illustrate the solvent’s power to release absorbed energy as heat to the sample material. Polyphenols are dipoles that can absorb microwave energy due to their hydroxyl groups; therefore MAE is a technique that can be used for the extraction of these compounds [96,97]. Aqueous acetone, ethanol, or their mixtures are employed to extract phenolic compounds using MAE [93]. As MAE is influenced by many factors, several statistical optimizations have been performed to determine the best operating conditions to extract different phenolics [95,98].

Xiao *et al.* [99] evaluated all the influential parameters mentioned above to extract flavonoids from *Radix astragali*. The selected conditions were 60–100% v/v aqueous ethanol, 10–40 mL solvent per g material, 5–30 min irradiation, 70–130 °C and 200–1000 W microwave power. The most effective extraction was achieved by applying 25 mL of 90% ethanol for 25 min at 110 °C under 1,000 W.

In other research, the effects of temperature, ethanol composition and time on the percent recovery of the anthraquinones extracted from *Morinda citrifolia* by MAE were determined [100]. The results revealed that MAE has the power to give the highest yield compared to other methods. The reported appropriate MAE conditions were 80% aqueous ethanol at a temperature of 60 °C for 30 min.

The above-mentioned results demonstrate the potential for new MAE methods to extract phenolic compounds from plant material when compared with other extraction method such as maceration, UAE and Soxhlet. Table 3 summarizes some other research on optimization of phenolic extraction using MAE.

**Table 3:** Optimized conditions for phenolic extraction from plant-based foods using MAE.

Sample	Analyte	Solvent	MAE time (min)	MAE temperature (°C)	MAE power (W)	Solvent/sample (mL/g)	Reference
Green Tea	Flavanol	Water	30	80	600	20	[101]
Tea	Polyphenols	Ethanol 60%	10	80	600	12	[102]
*Ipomoea batatas*	TPC ^a^	Ethanol 53%	2.05	--	302	30	[103]
*Phaseolus vulgaris*	TPC ^a^	Ethanol 50%	15	150	--	49	[104]
*Fagopyrum esculentum*	TPC ^a^	Ethanol 50%	15	150	--	50	[105]
*Visit vinifera*	TPC ^a^, Flavonoids	Methanol 100%	60	110	60	5	[36]
*Melilotus officinalis* (L.)	Coumarin	Ethanol 50%	5	50	100	20	[106]
Vanilla beans	Vanillin	Ethanol 70%	20	--	150	25	[107]
*Radix angelicae sinensis*	Ferulic acid	Ethanol 90%	9	--	850	6	[108]
*Saussurea medusa*	Flavonoids	Ethanol 80%	60	80	1200	50	[109]
Sorghum	Phenolic acids	2 M NaOH	0.45	190	1400	25	[110]
Spices	Phenolic acids	Ethanol 50%	18	50	200	20	[111]

^a^ Total phenolic content.

### 4.3. Ultrasound/Microwave Assisted Extraction (UMAE)

The coupling of two powerful radiation techniques (ultrasonic and microwave) is a new efficient approach to extract bioactive compounds. As mentioned earlier, MAE is a simple and rapid technique using dielectric mechanisms to heat samples and extract the plant bioactive compounds [92], whereas UAE forms cavitations, which increase mass transfer and improve penetration of the solvent into the sample [112]. Thus, ultrasound/microwave-assisted extraction (UMAE) is a powerful technique that can reduce extraction time, consume lower volumes of solvents and result in higher extraction yields than conventional extraction, MAE and UAE [113].

Lou *et al.* [114] applied microwaves with ultrasonic extraction (UAME) and maceration to extract phenolics from Burdock leaves. The final optimized UMAE method gave a phenolic yield of 9 mg/g while less than 0.5 mg/g was achieved using maceration.

In another study, the yields of flavonoids from *Spatholobus suberectus* obtained by UMAE were compared with MAE, UAE, Soxhlet and heated reflux extraction methods under optimized conditions. The highest yield obtained for UAME was after 7.5 min using 20 mL/g solvent-sample ratio, while for other extractions, optimum yields depended on a higher solvent-sample ratio (40–120 mL/g) and longer time (30–3,600 min) [115].

**Figure 2:**
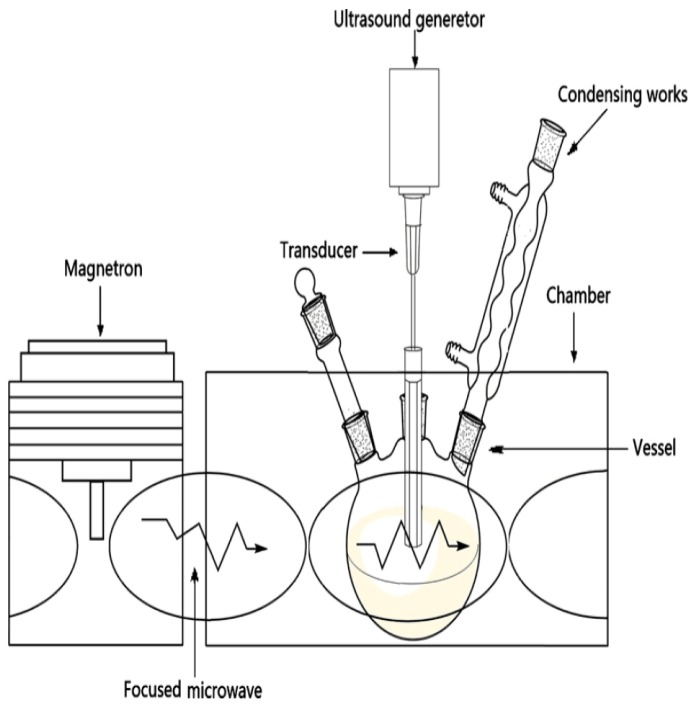
Schematic diagram of an apparatus for ultrasonic-microwave assisted extraction (UMAE) [115].

Tomato paste lycopene has been extracted using UMAE and UAE. The optimized time needed to give the highest yield of extract (97.4% lycopene) with UMAE was 367 seconds, whereas the corresponding time for UAE was 1,746 seconds and gave a lower yield (89.4% lycopene) [116]. These results above imply that UMAE is a more efficient extraction method than the other extraction techniques tested. A schematic diagram of an apparatus for UMAE is presented in Figure 2 [115].

Table 4 summarizes results using optimized UMAE to extract bioactive compounds from plant materials.

**Table 4:** Conditions for phenolic extraction from plant-based foods using UMAE.

Sample	Analyte	Solvent	Ultrasound power (W)	Microwave power (W)	UMAE time (s)	UMAE temp (°C)	Solvent/sample (mL/g)	Ref.
*Arctium lappa*	Caffeic acid	Ionic solution	50	400	30	--	20	[117]
*Spatholobus suberectus*	Flavonoids	Methanol 70%	50	300	450	80	20	[115]
Tomato	Lycopene	Ethyl acetate	50	98	367	--	10.6	[116]
Burdock leaves	Phenoliccompounds	Ethanol 70%	50	500	30	--	20	[114]
*Anoectochilu roxburghii*	Quercetin	Ethanol 50%	50	800	900	45	8	[118]

### 4.4. Supercritical Fluid Extraction (SFE)

Supercritical fluid extraction (SFE) is another environmentally friendly extraction technique, which can be a good alternative to conventional organic solvent extraction methods [1]. It may lower the requirement for toxic organic solvents, increase safety and selectivity, lower extraction time and facilitate separation of the extract from the supercritical fluids (SCF). Furthermore, degradation of extracted compounds can be avoided in the absence of air and light and the possibility of contaminating the sample with solvent impurities is much lower than in other methods [119,120]. The high capital investment for equipment is the main disadvantage of SFE. 

A SCF is a type of solvent that forms when the temperature and pressure of the fluid increase above its critical point [120]. The SCF generated has the penetration power of the gas form and density of the liquid form [96,121]. The usual SCF applied in SFE are methane, carbon dioxide, ethane, propane, ammonia, ethanol, benzene and water. Table 5 illustrates the critical temperature (Tc) and a pressure (Pc) of some SCFs. 

**Table 5:** Critical properties of commonly used SCFs.

Solvent	Pc (bar)	Tc (°C)	Density (g/mL)
Methane	46.41	−82.4	0.16
Carbon dioxide	73.87	31.2	0.47
Ethane	48.84	32.5	0.20
Propane	42.46	97.3	0.22
Ammonia	113.99	132.6	0.24
Ethanol	63.83	243.6	0.28
Benzene	48.94	289.1	0.30
Water	221.19	374.3	0.32

CO_2_ is the most commonly utilized SCF in SFE. It is chemically stable, has relatively low toxicity, is not flammable, is inexpensive and produces zero surface tension [122]. Furthermore, it has a mild critical temperature required for extraction of thermolabile compounds and is separated easily from the sample [123]. However, CO_2_ is non-polar and thus unsuitable for extraction of polar phenolic compounds. To cover this weakness and boost CO_2_ extraction power, the addition of polar co-solvents such as ethanol, methanol, ethyl acetate and acetone is recommended [119]. In the last decade, research has been conducted to optimize the extraction of phenolic compounds by SFE by varying pressure, temperature, extraction time, modifier and the solvent/ modifier mixture ratio [74,124,125]. For most phenolic materials, the highest yield was attained when the pressure was 50–600 bar, temperature 35–20 °C and time 5–180 min [126].

Different extraction methods including Soxhlet, MAE and UAE, as well as SFE, have been applied to determine total phenolic content of pomegranate seed oil. The different organic solvent extraction methods used in this study did not generate any significant differences in the total phenolics extracted, whereas the extracted oils from modified SFE gave a significantly higher yield of phenolic compounds [124]. In a study on oat bran, Holliday [127] reported that total phenolic content and antioxidant activity obtained under SFE conditions was higher than with MAE and conventional solvent extraction. However, the opposite was found for the amount of phenolics detected in the SFE extract [102]. The difference between SFE and MAE, as two of the more accepted techniques, may be due to different extraction times and temperatures, and the presence or absence of modifier solvent. Table 6 summarizes SFE conditions for extraction of phenolic compounds from some plant-based samples.

**Table 6:** SFE conditions for extraction of phenolic compounds from plant-based samples.

Sample	Target phenolic class	Temperature (°C)	Time (min)	Pressure (bar)	Modifier	Ref.
Elder berry and grape marc	Phenolic compounds	40	--	150, 350	Ethanol	[68]
*Theobroma cacao* hulls	Phenolic compounds	50	--	100, 200	Methanol and Acetone	[128]
Sweet basil	Phenolic compounds	35, 50	15, 30, 45, 60	100, 150, 200, 250, 300	H_2_O	[129]
*Baccharis dracunculifolia* leaves	Phenolic compounds	40, 50, 60	--	200, 300, 400	--	[130]
Guava seed	Phenolic content	40, 50, 60	120	100, 200, 300	Ethylacetate and Ethanol	[70]
Wheat germ	Phenolic content	40, 60	10, 60	148, 602	--	[125]
Pistachio hulls	Phenolic content	35, 45, 55	15, 25, 40	100, 200, 350	Methanol	[131]
Bupleurum roots	Phenolic content	40	--	50, 100, 150, 200	--	[132]
Bitter melon	Flavonoids	30, 40, 50	40, 50, 60	250, 300, 350	Ethanol	[133]
Spearmint leaves	Flavonoids	40, 50, 60	30, 60, 90	100, 200, 300	Ethanol	[134]
Pecah Kaca	Flavonoids	40, 50, 60	40,60,80	100, 150, 200	Ethanol	[135]
*Pueraria lobata*	Flavonoids	40, 50, 60	90	150, 200, 250	Ethanol	[136]

### 4.5. Subcritical Water Extraction (SCWE)

Another environmentally friendly extraction technique that has been utilized to efficiently isolate phenolic compounds is subcritical water extraction (SCWE) [137,138], also known as superheated water, pressurized water or hot liquid water extraction. The main advantages of SCWE over conventional methods are its simplicity, high extract quality, low extraction time and environmental friendliness due to water being used as the solvent [138]. With SFE, only non-polar compounds can be extracted from plant material using organic solvents as modifiers, and plant processing is likely to be more expensive than with SCWE [138].

Water becomes subcritical when the temperature is 100–347 °C applied under sufficient pressure (normally 10–60 bar) to preserve its liquid form (below 220 Bar) [139]. The dielectric constant of water reduces under subcritical conditions due to the breakdown of intermolecular hydrogen bonds. By adjusting parameters like pressure and temperature, subcritical water displays different dielectric constant values and polarity (*i.e.*, ethanol-water and methanol-water) [9,140]. Water at room temperature has high polarity and a dielectric constant close to 80. By applying suitable pressure to keep water in liquid form at 250 °C, the dielectric constant decreases to 27, which is similar to that of ethanol [141].

Treatment with SCWE has been shown to be sufficiently powerful to extract a wide range of polar to low-polar compounds such as phenolic acids from grape skin [142] and essential oils from coriander seeds [143]. For extraction of anthraquinones from *Morinda citrifolia*, the effectiveness of SCWE compared to that of other extraction methods, such as ethanol extraction in a stirred vessel, Soxhlet extraction and ultrasound-assisted extraction, has been studied [144]. The results indicated that SCWE extracts gave almost the same antioxidant activity as Soxhlet extracts, but SCWE extracts contained higher antioxidant activity than ethanol extracts and ultrasound-assisted extracts.

SCWE could be a good alternative industrial method to use for extraction of large amounts of phenolic compounds without toxic organic solvent residues. The products are ready to use as antioxidants for food products. Table 7 reports some recent work on the extraction of phenolics from plant materials using SCWE.

**Table 7:** Conditions for SCWE of phenolic compounds from plant-based materials.

Sample	Analyte	Temperature (°C)	Time (min)	Pressure (bar)	Solvent/sample (mL/g)	Ref.
Pomegranate seeds	Phenolic compounds	80–280	15–120	60	10–50	[145]
Cinnamon bark	Phenolic compounds	150,200	60	60	--	[146]
Potato peel	Phenolic compounds	100–240	30–120	60	--	[147]
Rice bran	Phenolic compounds	125–200	5	20	2.5	[148]
*Terminalia chebula*	Phenolic compounds	120–220	10–150	40	--	[149]
Bitter melon	Phenolic compounds	130–200	10–120	--	--	[150]
Oregano leaves	Phenolic compounds	25–200	15, 30	103.4	--	[151]
Green tea	Catechin and epicatechin	140–260	--	38–72	20	[152]

### 4.6. High Hydrostatic Pressure Extraction (HHPE)

Another novel technique that can be utilized to extract phenolics from plants is HHPE. This method utilizes non-thermal super-high hydraulic pressure (1,000–8,000 bar) and works on the basis of mass transport phenomena [153,154]. The pressure applied increases plant cell permeability, leading to cell component diffusivity according to mass transfer and phase behavior theories [153,155,156]. A main disadvantage of methods such as HHPE, SCWE and SFE is that expensive equipment is required; *i.e.*, a solvent transporting pump, a pressure vessel and system controller, and a collection device for the extract [157]. However, in the case of antioxidant extraction, in which products are in great demand and high purity of extract and processing efficiency are expected, the price of equipment might not play a critical role in selection of these methods [158].

HHPE involves creation of a huge pressure difference between the cell membrane interior and exterior and allows solvent to penetrate into the cell causing leakage of cell components [153,155]. HHPE can also cause cell deformation and protein denaturation, which can reduce cell selectivity and increase extraction yield [159].

HHPE is usually conducted at ambient temperature using different solvents from polar to non-polar, depending on the bioactive compounds to be extracted. The feasibility of HHPE to extract phenolic compounds from plant material is clearly demonstrated in some studies. Higher yields of phenolic compounds from *Maclura pomifera* fruits, anthocyanins from grape by-products and flavonoids from propolis have been obtained using HHPE compared with conventional extraction methods [72,153,160]. HHPE is also reported to be suitable to extract polyphenols from green tea leaves [159]. A higher yield of soluble polyphenols in the juice of cashew apples has been obtained using HHPE compared to other methods [161].

### 4.7. Other Extraction Methods

Pulsed electric field (PEF) processing is a non-thermal technique requiring low energy to increase cell membrane breakdown and mass transfer [162]. PEF can be operated continuously at room temperature and performed in a matter of seconds [163]. Such positive factors play an important role in minimizing quality deterioration of food compounds, especially bioactive materials [163]. Application of PEF to red cabbage, strawberry, must of tempranillo grapes, chardonnay grapes and merlot grapes increased the yield of total phenolics extracted [164,165,166,167,168]. In contrast, Turk *et al.* [169] reported a lower yield of phenolic compounds of apple juice extracted by PEF.

Accelerated solvent extraction (ASE) is an automated technique using common organic solvents to extract phenolics from plant materials [170]. ASE operates under nitrogen at high temperature and pressure, which helps the solvent penetrate rapidly into the plant cells and prevents degradation of phenolic compounds. Compared to conventional methods, the amount of solvent and extraction time are dramatically lower [171].

Sequential alkaline extraction is a method used to extract free and bound phenolic compounds from plant materials [105]. Free phenolics were extracted using water, pure organic or aqueous organic solvent under a nitrogen atmosphere in a water bath for 20 min. The solid residue was then hydrolyzed with NaOH for 1 h under N_2_ in the dark at room temperature. The alkaline extract was treated by HCl to reach pH 2, centrifuged, and the extract was used for the determination of bound phenolics. 

Enzymatic treatment of plant samples is another technique suitable for the liberation of phenolic compounds. Phenolics in plant materials largely appear to be linked with plant cell wall polysaccharides by both hydrophilic and hydrophobic bonds [172]. The addition of enzymes might disintegrate the phenolic-cell wall matrix bonds and enhance phenolic extraction [24,173,174]. Recently, enzymatic hydrolysis using a combination of pectinase, cellulase and hemicellulase was shown to enhance phenolic extraction from raspberry solid waste [175]. Maier *et al.* [176] developed the application of enzymes to phenolic extraction from grape pomace. Kapasakalidis *et al.* [61] reported that commercial cellulose enzyme preparations promote the extraction of polyphenols and anthocyanins from black currant pomace. In other research, a comparison of the application of three different types of enzyme preparations including α-amylase, Viscozyme L, and Ultraflo L was conducted on *Ipomoea batatas* (sweet potato) stem [177]. Ultraflo L and Viscozyme L facilitated phenolic recovery and resulted in a higher yield of ferulic acid and vanillic acid, respectively, in the extract. Hong and Van Veit [178] compared UAE techniques and enzyme-assisted extraction of phenolic compounds from acerola fruit, finding, in contrast, a higher yield of phenolics using novel UAE methods than enzymatic extraction.

In summary, MAE, UMAE, SFE and pressurized solvent extraction methods such as SCWE and HHPE are fast and efficient unconventional extraction methods developed for extracting analytes from plant matrices. They are emerging as good alternatives to conventional extraction methods, mainly due to lack of need for organic solvents and relatively low extraction times. Due to differing availabilities of instruments in analytical laboratories, sample complexity, solvent types, extraction time and temperature, sample-solvent ratio, type of target extract and many other factors, selection of extraction methods or even placing them in order of their advantages and disadvantages is difficult. For research-scale extraction, however, UMAE is highly recommended for many plant-based samples because of its effectiveness and relatively low cost.

## 5. Quantification of Phenolics

Despite a very large number of published investigations, quantification of various phenolic structural groups remains difficult [120,179]. Thus there is great scope for developing quantification methods based on the type of phenolic group. [180]. High performance liquid chromatography (HPLC) and gas chromatography (GC), or their combinations, with mass spectrometry are the two most commonly applied methods to quantify phenolic compounds. Other relevant techniques include spectrophotometric assays [28]. 

### 5.1. Spectrophotometric Assays

Spectrophotometry is one of the relatively simple techniques for quantification of plant phenolics. The Folin-Denis and Folin-Ciocalteu methods were the two widely used specrophotometric assays to measure total phenolics in plant materials for many years [181,182]. Both methods are based on a chemical reduction involving reagents containing tungsten and molybdenum [24]. The products of this reduction in the presence of phenolic compounds have a blue color with a broad light absorption spectrum around 760 nm. The reagents for both methods do not react specifically with only phenols but also with other substances like ascorbic acid, aromatic amines and sugars [183].

Total phenolic quantification, total flavonoids, proanthocyanidin (condensed tannin) and hydrolysable tannin can also be estimated by colorimetric methods. Methanolic or ethanolic extracts of plant phenols mixed with AlCl_3_ allow measurement of total flavonoids in the range 410–423 nm [184,185].

Vanillin and dimethylaminocinnamaldehyde (DMCA) assays are used to determine the level of proanthocyanidins [28]. These methods can provide information about the degree of polymerization and the hydroxylation pattern and stereochemistry of flavan-3-ol subunits [186,187]. Catechin is usually used as a standard in the vanillin method and as a result may lead to the over-estimation of proanthocyanidins. The accuracy of the DMCA assay to quantify proanthocyanidins is also questionable [188].

The butanol-HCl and bovine serum albumin (BSA) methods are the other proanthocyanidin determination techniques. The butanol-HCl method is based on cleavage of interflavonoid bonds in proanthocyanidin using hot acid, followed by an auto-oxidation reaction to convert flavan-3-ols to anthocyanidin. The red extract formed has a maximum absorbance at around 550 nm [189]. In the BSA method, insoluble tannin-protein complexes are precipitated by treating samples with bovine serum albumin. The tannin-protein complex is dissolved in alkaline sodium dodecyl sulphate-triethanolamine solution and reacted with ferric chloride solution to form a violet complex with a maximum absorbance at 510 nm [190].

A validated method to quantify proanthocyanidin in grape extract based on precipitation of proanthocyanins using methyl cellulose has been published. The proanthocyanidins form an insoluble polymer after reacting with methyl cellulose [191]. In this method, proanthocyanidin concentration can be checked by measuring absorbance before and after methyl cellulose treatment [192]. Hydrolysable tannins can be evaluated using the potassium iodate method, rhodanine method and sodium nitrite method [1]. Among them, potassium iodate is the most popular method for screening samples. A red color with a maximum absorbance of 500–550 nm appears due to the reaction of methyl gallate and potassium iodate [187]. The rhodanine and sodium nitrite methods can also be used to determine hydrolysable tannins based on the presence of gallic and ellagic acid in the sample, respectively [193,194]. Another spectrophotometric method used to quantify flavonones and dihydroflavonols is based on their interaction with acidic 2,4-dinitrophenylhydrazine [195]. Pinocembrin is the standard used in this assay and the absorbance is measured at 486 nm [196].

Anthocyanins constitute the other main class of phenolic compounds measured by spectrophotometry. The main spectrophotometric assays applied to determine anthocyanins were reviewed by Giusti and Wrolstad [197]. Quantification of anthocyanin takes place in weak acidic media in the wavelength range 490–550 nm [198]. Colorimetric techniques to determine phenolics are simple and economical but only give an estimation of phenolic compound concentrations above a certain minimum level and do not quantify phenolics individually; however, these techniques can be useful for quick and relatively inexpensive screening of numerous samples [120].

### 5.2. Gas Chromatography

Gas chromatography (GC) is another technique applied for the separation, identification and quantification of phenolic compounds such as phenolic acids [199], condensed tannins [200] and flavonoids [201]. The major concerns of GC analysis, that are not applicable to HPLC techniques, are the derivatization and volatility of phenolic compounds. With GC, quantification of phenolics from food matrices may involve clean-up steps such as lipid removal from the extract, release of phenolics from the glycoside and ester bonds in enzymatic [202], alkaline [203] and acidic [204] media and chemical modification steps, such as transformation to more volatile derivatives [180]. 

There are a several types of reagents used to modify and create volatile derivatives. Ethyl and methyl chloroformate, diazomethane and dimethyl sulfoxide in combination with methyl iodate are used to make methyl or ethyl esters of phenolics. However, in some studies, substantial confusion may occur due to the presence of methyl esters in a natural form [205,206,207]. Another generation of reagents, which have advantages in the creation of volatile compounds, are the trimethylsylil family of compounds, such as trifluoroacetymide, *N*-(*tert*-butyldimethylsilyl)-*N*-methyltrifluoroacetamide and trimethylsilyl derivatives [120,208]. The silylation reaction is simple, free of unwanted side products and produces tremendously volatile products with no interference with the analysis [209]. Silyl derivatization is thus a very good option to identify phenolic compounds but more research is needed on identification of silyl derivatives [201].

Fused silica capillaries of 30 m lengths, with internal diameters of 25–32 μm and stationary phase particle size of 0.25 μm are the most common columns used for phenolic quantification in GC techniques. There are exceptions, however, such as the column used by Shadkami *et al.* [200] with 15 m length and 10 μm film thickness. 

The use of a flame ionization detector (FID) is the most common method to detect phenolics but mass spectroscopy (MS) has become widespread recently [209]. GC provides more sensitivity and selectivity when combined with mass spectrometry [120]. For instance, the difficulties of flavonoid glycoside evaluation by conventional GC were solved when high-temperature–high-resolution GC–MS was applied [210]. Another study indicated that GC-MS analysis of phenolic and flavonoid standards was more efficient than that of HPLC, providing a fast analysis with better resolution and baseline separation of all standards with minimum co-elution [211]. Some of the gas chromatographic techniques for the analysis of phenolic compounds are presented in Table 8.

### 5.3. High Performance Liquid Chromatography

HPLC is the preferred technique for both separation and quantification of phenolic compounds [28]. Various factors affect HPLC analysis of phenolics, including sample purification, mobile phase, column types and detectors [24]. In general, purified phenolics are applied to an HPLC instrument utilizing a reversed phase C18 column (RP-C18), photo diode array detector (PDA) and polar acidified organic solvents [120]. Several reviews are available on the application of HPLC and the quantification of phenolics [24,209,212,213,214]. Normally, HPLC sensitivity and detection is based on purification of phenolics and pre-concentration from complex matrices of crude plant extracts.

**Table 8:** Summary of GC conditions to detect molecules belonging to phenolic classes.

Sample	Derivatization	Detected phenolics	Detection	Chromatographic assay details	Ref.
Guarana	Dried phenolic extract derivatized with a mixture of hexamethyldisiloxane and dimethylchlorosilane in pyridine	3-Hydroxybenzoic acid, benzoic acid, gallic acid, syringic acid, isovanillic acid, protocatechuic acid, catechin, caffeine, epicatechin, quercetin	GC–MS	Zebron ZB-5 ms fused silica capillary column (30 m × 0.25 mm I.D. × 0.25 μm film thickness); Oven temperature: 150 °C held for 5 min, to 295 °C at 3 °C/min, held for 18 min; Injector temperature: 300 °C; Carrier gas: helium flow at 1 mL/min; Ion source temperature: 200 °C; Transfer line temperature: 290 °C	[215]
Mirabelle plums	Dried phenolic extractderivatized with *N*,*O*-Bis(trimethylsilyl)trifluoro-acetamide	Benzoic acid, *p*-hydroxybenzaldehyde, *p*-hydroxybenzoic acid, vanillin, 3,4-dihydroxybenzoic acid, vanillic acid, gallic acid, syringaldehyde, syringic acid, coniferyl aldehyde, 3,5-dimethoxycinnamaldehyde, dehydrodiconiferyl aldehyde, guajacyl-glycerin-coniferyl aldehyde, guajacyl-glycerin-coniferyl aldehyde	GC–MS	HP 5MS capillary column, (30 m × 0.25 mm I.D × 0.25 μm film thickness). Oven temperature: 100–270 °C at 4 °C /min, held for 20 min; Injector temperature: 250 °C; Helium flow at 0.9 mL/s; Ion source temperature: 230 °C; Transfer line temperature: 280 °C	[216]
Guava bagasse, Cabernet Sauvignon, Pinot Noir, and Isabella grape marcs wastes	---------------------	Succinic acid, azelaic acid, syringic acid, *p*-coumaric acid, gallic acid, ferulic acid, caffeic acid, epicatechin, quercetin, myricetin	GC–MS	RTX 5MS capillary column (30 m × 0.25 mm ID × 0.25 μm film thickness); Oven	[199]
Cranberry	Dried phenolic extract derivatized with a mixture of *N*,*O*-Bis(trimethylsilyl)-trifluoroacetamide and 1% trimethylchlorosilane in pyridine	Benzoic acid, *o*-hydroxybenzoic acid, trans-cinnamic acid, *m*-hydroxybenzoic acid, *p*-hydroxybenzoic acid, *p*-hydroxyphenyl acetic acis	GC-MS	Temperature: 80 °C for 1 min, to 250°C, at 20°C/min, held 1 min, to 300°C at 6°C/min, held 5 min, to 310°C at 15 °C/min held 10 min, to 320 °C at 20°C/min, held 10 min; Injector temperature: 280 °C; Transfer line temperature: 280 °C. DB-5 fused-silica capillary column (30 m × 0.32 mm ID × 0.25 μm film thickness)	[217]
Saffron corms	Dried phenolic extract derivatized with a mixture of *N*-methyl-*N*-(trimethylsilyl) trifluoroacetamide and iodotrimethylsilane	Acetic acid, *o*-phthalic acid, 2,3-dihydroxy-benzoic acid, vanillic acid, *o*-hydroxy-cinnamic acid, 2,4-dihydroxy-benzoic acid, *p*-coumaric acid, ferulic acid, caffeic acid, sinapic acid, epicatechin, catechin. Quercetin, myricetin, *m*-methylbenzoic acid. catechol, vanillin, salicylic acid, cinnamic acid, *p*-hydroxybenzoic acid, syringic acid, *p*-coumaric acid, gallic acid, *t*-ferulic acid, caffeic acid, gentisic acid	GC-MS	Oven temperature: 80 °C for 1 min, to 220 °C, at 10 °C/min, to 310 °C, at 20 °C/min, held 6 min; Injector temperature: 280 °C; Detector temperature: 305 °C; Transfer line temperature: 280 °C. DB-5 capillary column (30 m × 0.25 mm ID × 0.25 μm film thickness); Oven temperature: 140 °C for 2 min, to 270 °C at 5°C/min	[218]
Mangosteen fruit	Dried phenolic extract derivatized with *N*,*O*-bis(trimethylsilyl)acetamide	Hydroxybenzoic acid, protocatechuic acid, vanillic acid, caffeic acid, *p*-coumaric acid, ferulic acid, *p*-hydroxyphenylacetic acid, 3,4-dihydroxymandelic, cinnamic acid	GC-MS	Held 20 min; Injector temperature: 270 °C; Transfer line temperature: 270 °C. SPB-1 silica-fused capillary column (30 m × 0.25 mm ID × 0.25 μm film thickness); Oven temperature: 120 °C held 2 min, to 260°C at 20 °C /min , held 10 min; Injector temperature: 240 °C; Helium flow at 28 cm^3^/min; Transfer line temperature: 240 °C.	[208]
Green tea	Dried phenolic extract derivatized with trimethyl-sulfonium hydroxide and trimethylsilyl diazomethane	Catechin, epicatechin, epigallocatechin, gallocatechin, kaempferol, quercetin	GC-MS	A ZB-5HT Inferno capillary column (15 m × 0.32 mm ID × 0.10 μm film thickness); Oven temperature: 100°C held for 5 min, to 375°C at 20°C/ min, held for 5 min; Injector temperature: 350°C; Transfer line temperature: 300°C	[200]
Various plant extracts	Dried phenolic extract derivatized with a mixture of trimethylchlorosilane and *N*,*O*-bis(trimethylsilyl)-trifluoroacetamide with dimethyldichlorosilane in toluene and dimethyldichlorosilane	Gallic acid, *p*-hydroxybenzoic acid, gentisic acid, *p*-coumaric acid, vanillic acid, ferulic acid, syringic acid, catechin	GC-MS	CP-Sil 8 capillary column (30 m × 0.32 mm ID × 0.25 μm film thickness)	[195]
Propolis	Dried phenolic extract derivatized with *N*,*O*-Bis(trimethylsilyl)trifluoro-acetamide	quercetin, apigenin, naringenin, luteolin, caffeic acid, epicatechin, rutin, hydroxytyrosol. Ethyl hydrocinnamate, hydrocinnamic acid, inositol, cinnamic acid, ferulic acid, caffeic acid, pinostrobin	GC-MS	Oven temperature: 70 °C, to 135 °C at 2 °C /min, held for 10 min, to 220 °C at 4°C /min, held for 10 min, to 270 °C at 3.5 °C/min, held for 20 min; Injector temperature: 280 °C; Transfer line temperature: 290 °C. Borosilicate capillary column (20 mm × 0.3 mm ID × 0.1 μm)	[219]

**Table 9:** Summary of recent HPLC conditions for plant and food phenolic classes.

Sample	Phenolic class	Column/Detector	Solvent/ Flow rate/ injection volume	Temperature (°C)/Detection time (min)	Ref.
Mangosteen pericarp	Gallic acid, gentisic acid, protocatechuic acid, gentisic acid, 4-hydroxybenzoic acid, veratric acid, vanillic acid, caffeic acid, syringic acid, *p*-coumaric acid, sinapic acid, ferulic acid, *t*-cinnamic acid catechin, epicatechin	Bondapak C18 column (300 mm × 3.9 mm ID × 5 μm)/ PDA ^b^, ESI-MS ^e^	Water : methanol : acetic acid (85:14:1); Flow rate: 1.0 mL/min; Injection volume: 20 μL	Ambient/ 45	[220]
Mulberry fruit	Cyanidin 3-*O*-rutinoside, cyanidin 3-*O*-glucoside, pelargonidin 3-*O*-glucoside, pelargonidin 3-*O*-rutinoside	RP C18 column (250 mm × 4.6 mm ID, 5 μm)/ PDA ^b^, ESI-MS ^e^	A: water containing 0.1% TFA (trifluoroacetic acid); B: acetonitrile containing 0.1% TFA; Elution profile: 0–2 min, 10% B; 2–35 min, 10–90% B; 35–40 min, 90–100% B; 40–60 min, 100% B/ Flow rate: 1.0 mL/min; NM^a^	Ambient/ 60	[221]
Fruit juice	Cyanidin, peonidin, delphinidin, petunidin, malvidin, pelargonidin	ODS-3 column (250 mm × 4.6 mm ID × 5 μm)/ PDA ^b^	A: acetonitrile; B: water containing 10% acetic acid and 1% phosphoric acid; Elution profile: 25 min, 2–20% A; 5 min, 20–40%; Flow rate: 1.0 mL/min; Injection volume: 25 μL	NM ^a^/50	[222]
*Maytenus aquifolium* and *Maytenus ilicifolia* Leaves	Quercetin, kaempferol derivatives, rutin	Supelcosil C8 and C18 (250 mm × 4.6 mm ID × 5 μm) column/ PDA ^b^	A: water containing 2.0, 2.5 or 3.0% formic acid or 0.3% trifluoroacetic acid; B: acetonitrile or methanol; Various elution profiles; Flow rate 1.0 mL/ min/ Injection volume: 10 μL	35/ Different detection times	[223]
Apple	Gallic acid, chlorogenic acid, catechin, epicatechin, procyanidin, phloridzin, cyanidin 3-galactoside, quercetin 3-rutinoside, quercetin 3-galactoside, quercetin 3-glucoside, quercetin 3-rhamnoside	RP C18 (250 mm × 4.6 mm ID × 4 μm) column/ PDA ^b^	A: water containing 1% TFA, B: ACN containing 1% TFA; Elution profile: 0–10 min, 10% B; 10–45 min, 10–20% B; 45–50 min, 20–50% B; 50–55 min, isocratic 50%; 55–60 min, 50–10% B. Flow rate: 1 mL/min. Injection volume: 10 μL	40/ 60	[224]
Medicinal plants	Cyanidin glucoside, pelargonidin glucoside, gallocatechin-catechin gallate, afzelechin–catechin dimer, gallocatechin catechin gallate, ferulic acid glucoside, rutin, naringenin-7-*O*-rutinoside	RP C18 (250 mm × 4.6 mm ID × 5 μm) column/ PDA ^b^, ESI- MS ^e^	A: water containing 1% formic acid, B: acetonitrile; Elution profile: 30 min, 90–75% A; 30–45 min, 75–40% A; Flow rate: 1 mL/min; Injection volume: 20 μL	25/ 45	[225]
Food samples	Monomeric, dimeric and trimeric procyanidins, catechin, epicatechin	RP 18 (250 mm × 2 mm ID × 5 μm) column/ PDA ^b^, FLD ^d^, ESI-MS/MS	A: water containing 0.1% formic acid; B: acetonitrile containing 0.1% formic acid; Elution profile: 0–10 min, 10% B; 10–30 min, 15% B; 30–65 min, 40% B. Flow rate: 300 μL/min; Injection volume: 20 μL	25/ 30	[226]
Oregano	Quercetin, fisetin, kaempferol, luteolin, apigenin, eriodictyol, hesperetin, taxifolin, (+)-catechin, (-)-epicatechin	Hypersil C18 ODS (250 mm × 4.6 mm ID × 5 μm) column/ PDA ^b^, ESI-MS-MS	A: water; B: methanol; C: acetonitrile, each containing 0.2% trifluroacetic acid; Elution profile: Initial, 90% A, 6% B, 4% C; 5 min, 85% A, 9% B, 6% C; 5–35 min, 71% A, 17.4% B, 11.6% C; 35–95 min, 0% A, 85% B, 15% C; Flow rate: 1 mL/min; NM ^a^	30/ NM ^a^	[51]
Lotus leaves	Myricetin 3-*O*-glucoside, quercetin 3-*O*-arabinopyranosyl, quercetin 3-*O*-glucuronide, kaempferol 3-*O*-galactoside, astragalin, isorhamnetin 3-*O*-glucoside, kaempferol 3-*O*-glucuronide, quercetin	C18 (150 mm × 4.6 mm ID × 3.5 μm) column/ PDA ^b^, ESI-MS ^e^	A: water containing 0.5% formic acid; B: acetonitrile containing 0.1% formic acid; Elution profile: 0–10 min, 12% B; 10–32 min, 12–20% B; 32–40 min, 20–30% B; 40–48 min, 30–60% B; 48–49 min, 60–12% B; 49–53 min 12% B; Flow rate: 0.6 mL/min; NM ^a^	30 /53	[227]
Bilberries and Blueberries	Delphinidin-3-*O*-glucopyranoside, delphinidin-3-*O*-galactopyranoside, cyanidin-3-*O*-arabinopyranoside, malvidin-3-*O*-arabinopyranoside, petunidin-3-*O*-galactopyranoside	C18 (250 mm × 4.6 mm ID × 3 μm) column/ UV-VISc	A: acetonitrile: water: formic acid (87/3/10); B acetonitrile: water: formic acid (50/40/10); Elution profile: 0–20 min, 2%–14% B; 20–40 min, 14% B; 40–50 min, 15% B; 50–55 min, 19% B; 55–65 min, 20% B/ Flow rate: 0.5 mL/min; Injection volume: 20 μL	Ambient/ 65	[228]
Persian walnut	3-caffeoylquinic, 3-*p*-coumaroylquinic, 4-*p*-coumaroylquinic acid, quercetin 3-galactoside, quercetin 3-arabinoside, quercetin 3-xyloside, quercetin 3-rhamnoside, quercetin 3-pentoside, kaempferol 3-pentoside	LiChroCART RP C 18 (250 mm × 4 mm ID × 5 μm)/ PDA ^b^, MS-MS	A: water containing 0.1% TFA; B: methanol; Elution profile: 30 min, 30–50% B; 30–32 min, 70% B; 32–33 min, 80% B, 33–35 min, 80% B; Flow rate: 1 mL/ min; Injection volume: 5 μL	NM ^a^/35	[229]
Rye grain	Sinapic acid, syringic acid, vanillic acid, ferulic acid, caffeic acid, *p*-hydroxybenzoic acid, protocatechuic acid, *p*-coumaric acid, ferulic acid dehydrodimers	Inertsil ODS-3 (150 mm × 4.0 mm ID × 3 μm/ PDA ^b^	A: 50 mM H_3_PO_4_ (pH 2.5) B: acetonitrile; Elution profile: 0–5 min, 95% A; 5–17 min, 95–85% A; 17–40 min, 85–80% A; 40–60 min, 80–50% A; 60–65 min 50% A. Flow rate: 0.7 mL/min; Injection volume: 10 μL	35/ 67	[230]
Pomegranate juices	Delphinidin 3,5-diglucoside, cyanidin 3,5-diglucoside, delphinidin 3-glucoside, pelargonidin 3,5-diglucoside, ellagic acid	RP C18 Nucleosil (125 mm × 5.0 mm ID × 5.0 μm) column/ UV-VIS ^c^	A: water containing 2.5% acetic acid; B: methanol containing 2.5%, acetic acid; Elution profile: 0–5 min, 100% A; 5–15 min, 90%; 15–45 min, 50% A; 45–55 min, 100% A. Flow rate: 1.0 mL/min; Injection volume: 50 μL	NM ^a^/55	[231]
Orange juice	Gallic acid, protocatechuic acid, *p*-hydroxybenzoic acid, vanillic acid, caffeic acid, chlorogenic acid, *p*-coumaric acid, ferulic acid, sinapic acid, narirutin, naringin, hesperidin, neohesperidin, didymin	Ultrasphere ODS (250 mm× 4.6 mm ID × 5 μm) column/ UV-VIS ^c^	A: water containing 5% formic acid; B: acetonitrile/solvent A (60:40; v/v); Elution profile: 0-10 min, 0% B; 10–40 min, 0–5% B; 40–58 min, 5–15% B; 48–62 min, 15–25%, 62–93 min, 25–50% B; 93–96 min, 50–100% B; Flow rate: 1.0 mL/min; NM ^a^	25/ 96	[232]
Quinoa	Apigenin-7-methyl ether, 1-*O*-galloyl-β-d-glucose, protocatechuic acid 4-*O*-glucoside, vanillic glucoside, penstebioside, ferulic acid 4-*O*-glucoside, ethyl-m-digallate, gallocatechin, quercetin, kaempferol, rutin	Kinetex C18 (100 mm × 4.6 mm ID × 2.6 μm) column/ PDA ^b^, ESI-MS ^e^	A: water containing 1% acetic acid; B: acetonitrile/solvent A (40:60; v/v); Elution profile: 0–3.5 min, 2% B; 3.5–4.5 min, 2–6%; 4.5–6 min, 6–10% B; 6–7.5 min, 10–17%; 7.5–13 min, 17–36% B; 13–14 min, 36–38.5% B; 14–19 min, 38.5–60% B; 19–24 min, 60–100% B; 24–30 min, 100% B; 30–32 min, 100–2% B; Flow rate: 0.8 mL/min; Injection volume: 10 μL	25/ 30	[233]
Pine needle	Catechin, proanthocyanidins	SupelcoSil LC18 (250 mm × 4.6 mm ID × 5 μm) column/ UV ^c^	A: acetonitrile; B: water containing 0.3% phosphoric acid; Elution profile: 0–35 min, 10–20% A; 35–55 min, 20–90% A; Flow rate: 0.7 ml/min; Injection volume: 10 μL	NM^a^/47	[234]
Apricot fruit	*p*-aminobenzoic acid, chlorgenic acid, caffeic acid, protocatechuic acid, ferulic acid, rutin, resveratrol, quercetin	Gemini C18 (150 mm × 4.6 mm ID × 3 μm) column/ UV-VIS ^c^	A: citric acid (75 mM); B: ammonium acetate (25 mM); Elution profile: 0–1 min, 5% B; 1–4 min, 5–6% B; 4–20 min, 6–25% B; 20–30 min, 25–100% B; 30–36 min, 100% B; 36–38 min, 100–5% B; 38–45 min, 5% B; Flow rate: 1.0 mL/min; Injection volume: 20 μL	35/45	[235]
Sage tea	Carnosic acid, epirosmanol, luteolin-rutinoside, salvianolic acid, apigenin-glucuronide, rosmarinic acid, apigenin-rutinoside, luteolin-rutinoside, luteolin-7-*O*-glucoside, monohydroxy benzoic acid, luteolin-diglucuronide, caffeic acid, caffeoyl-fructosyl-glucose, coumaroyl-hexose, protocatechuic acid	RP C18 (150 mm × 2.1 mm ID × 1.7 μm) column/ PDA ^b^, MS-MS	A: water containing 0.1% formic acid; B: acetonitrile containing 0.1% formic acid; Elution profile: 0–14 min, 4–27% A; 14–28 min, 27–59.7% A; 28–28.2 min, 59.7–100% A; 28.2–30.5 min, 100% A; 30.5–31 min, 100–4% A; 31–34 min, 4% A; Flow rate: 0.4 mL/min; Injection volume: 3 μL	40/ 28	[236]
Almond skin	Quercetin-3-*O*-glucoside, isorhamnetin-3-rutinoside, kampferol-3-rutinoside, naringenin-7-*O*-glucoside, isorhamnetin-3-glucoside, *p*-hydroxybenzoic acid, naringenin, protocatechuic acid, vanillic acid	RP C18 (50 mm × 2 mm ID, × 2.5 μm) column/ ESI-MS ^e^	A: water containing 0.1% formic acid. B: acetonitrile containing 0.1% formic acid; Elution profile: 0–9.5 min, 1–100% B; Flow rate: 0.5 mL/min; Injection volume: 5 μL	35/9.5	[237]
Burdock leaves	Quercetin, cynarin, benzoic acid, quercitrin, caffeic acid, luteolin, chlorogenic acid, *p*-coumaric acid, rutin, arctiin	BEH C18 (150 mm × 2.1 mm ID × 1.7 μm) column/ PDA, ESI-MS-MS	A: water containing 0.1% formic acid; B: acetonitrile/methanol (20/80); Elution profile: 0–10 min, 10–30% B; 10–20 min, 30–50% B; 20–23 min, 50–70% B; 23–25 min, 70–10% B; Flow rate: 0.28 mL/min; NM ^a^	NM ^a^/25	[114]
Grape extract	Malvidin glucoside, delphinidin glucoside, cyanidin glucoside, petunidin glucoside, peonidin glucoside, malvidin acetylglucoside, delphinidin acetylglucoside, cyanidin acetylglucoside, petunidin acetylglucoside, peonidin acetylglucoside, malvidin coumarylglucoside	Zorbax SB-C18 (50 mm × 2.1 mm ID × 1.8 μm) column /PDA ^b^, MS-MS	A: water containing 10%; B: acetonitrile; Elution profile: 0–1.5 min, 10–13% B; 1.5–4.5 min, 13-15% B; 4.5–7.5 min, 15–22% B; 7.5–15 min, 22% B; Flow rate: 0.2 mL/min; Injection volume: 1 μL	NM ^a^/15	[238]
Stem Bark of *Acacia confusa*	(+)-catechin, (−)-epicatechin, 4β-(2-aminoethylthio) catechin, 4β-(2-aminoethylthio) epicatechin	Hypersil ODS (250 mm × 4.6 mm ID × 2.5 μm) column/ ESI-MS ^e^	A: water containing 0.5 % trifluoroacetic acid; B: acetonitrile containing 0.5% trifluoroacetic acid; Elution profile: 0–5 min, 3% B; 5–15 min, 3%–9% B; 15–45 min, 9%–16% B; 45–60 min, 16%–60% B; Flow rate: 1 mL/min; NM ^a^	Ambient/NM ^a^	[239]
Lettuce	Caffeoyltartaric acid, *p*-coumaroyltartaric acid, caffeoylquinic acid, chlorogenic acid, *p*-coumaroylquinic acid, caffeoylmalic acid, dicaffeoyltartaric acid, chicoric acid, *p* -coumaroylcaffeoyltartaric acid, di-*p* -coumaroyltartaric acid, quercetin-3-*O*-glucuronide, 3,5-dicaffeoylquinic acid, quercetin malonylglucoside	HSS T3 (100 mm × 2.1 mm ID × 1.8 μm)column/ PDA ^b^, ESI-MS ^e^	A: water:methanol:formic acid (94.9:5.0:0.1); B: methanol:water:formic acid (60.0:39.9:/0.1); Elution profile: 0–30 min, 100–50% A; Flow rate: 0.5 mL/min; Injection volume: 10 μL	35/NM ^a^	[240]
Cocoa and Chocolate products	Catechin, epicatechin	diol-based (250 mm × 4.6 mm ID × 5 μm) /FLD ^d^, MS	A: acetonitrile:acetic acid (98:2); B: methanol:water:acetic acid (95:3:2); 0–35 min, 100–60% A; 35–39 min, 60% A; 39–41 min, 60–0% A; 41–47 min, 0.0% A; 47–51 min, 0–100% A; Flow rate: 1.0 mL/min; Injection volume: 10 μL	30/ 51	[241]
Wild mushroom	Benzoic acids, *p*-hydroxybenzoic, protocatechuic, vanillic, cinnamic, *p*-coumaric acids	Spherisorb RP C18 (150 mm × 4.6 mm ID × 3 μm) column/ PDA ^b^, ESI-MS ^e^	A: water containing 2.5% acetic acid; B: acetic acid 2.5%: acetonitrile (90:10); C: acetonitrile; Elution profile: 10 min, 100% A; 10–20 min, 50% A and 50% B; 20–35 min, 100% B; 35–45 min, 90% B and 10% C; 45–55 min, 70% B and 30% C; 55–60 min, 50% B and 50% C; 60–65 min, 20% B and 80% C; 65–70 min, 100% A; Flow rate: 0.50 mL/min; NM ^a^	25/NM ^a^	[242]
Cocoa, apple	Quercetin, phloridzin, clovamide, *p*-coumaroylquinic acid, caffeoylquinic acid, quercetin-3-*O*-galactoside, quercetin-3-*O*-arabinoside, quercetin-3-*O*-xyloside, dideoxyclovamide, quercetin-3-*O*-rhamnoside	BEH C18 (50 mm × 2.1 mm ID× 1.7 μm) column/ UV ^c^, FLD ^d^, ESI-MS ^e^	A: water containing 0.1 %formic acid; B: acetonitrile; Elution profile: 0–0.25 min, 2% B; 0.25–10.70 min, 2–18% B; 10.70–18 min, 18–25% B; 18–20.70 min, 25–100% B; 20.70–22.5 min, 100% B; Flow rate: 0.80 ml/min; Injection volume: 2 μL	50/22.5	[243]
Bean	Ferulic acid, *p*-coumaric acid, sinapic acid, caffeic acid	RP C18 Luna (150 mm × 4.6 mm ID × 5 μm) column/ PDA ^b^	A: water containing 0.1% formic acid; B: methanol; Elution profile: 0–50 min, 5–30% B; 50–65 min, 30% B; 65–75 min, 30–100% B; Flow rate: 0.7 mL/min; NM ^a^	25/65	[244]
Green tea, green coffee, grapefruit	Catechin, epigallocatechin gallate, epicatechin gallate, epicatechine, gallocatechin, catechin gallate, gallic acid, caffeine	RP C18 Atlantis (100 mm × 4.6 mm ID × 3 μm)/ UV	A: water containing 0.1% formic acid; B: methanol containing 0.1% formic acid; Elution profile: 0–5 min, 10% B; 5–14 min, 10–20% B; 14–20 min, 20–50% B; 20–22 min, 50–90% B; 22–26 min, 90% B; 26–30 min, 90–10% B; Flow rate: 0.5 mL/min; Injection volume: 20 μL	25/NM	[245]

^a^ Not mentioned; ^b^ Photodioide array detector; ^c^ Ultraviolet/ visible detector; ^d^ Fluorimetric detector; ^e^ Electroscopy ionization mass spectroscopy.

The purification stage includes removing the interfering compounds from the crude extract with partitionable solvents and using open column chromatography or an adsorption-desorption process. Sephadex LH-20, polyamide, Amberlite, solid phase extraction (SPE) cartridges and styrene–divinylbenzene (XAD 4, XAD16, EXA-90, EXA 118, SP70), acrylic resins (XAD-7, EXA-31) are examples of regularly applied materials to purify phenolics from crude sample extracts [246,247,248,249]. However, in most studies, SPE is used for purification and partial concentration prior to separation using HPLC [58,250,251].

Acetonitrile and methanol, or their aqueous forms, are the dominant mobile phases utilized in HPLC quantification of phenolics [220,221,223]. In some cases, ethanol, tetrahydrofuran (THF) and 2-propanol have been used [252,253,254]. Attention is recommended to maintain the pH of the mobile phase in the range pH 2–4 to avoid the ionization of phenolics during identification. Therefore, aqueous acidified mobile phases predominantly contain acetic acid but formic and phosphoric acids or phosphate, citrate and ammonium acetate buffers at low pH are also reported [24,222,223]. A gradient elution system is more commonly applied than an isocratic elution system [209].

Appropriate column selection is the other critical factor in identifying phenolics. Generally, based on the polarity, different classes of phenolics can be detected using a normal phase C18 or reversed phase (RP-C18) column 10–30 cm in length, 3.9–4.6 mm ID and 3–10 μm particle size [209,255]. However, new types of columns (monolithic and superficially porous particles columns) from 3–25 cm length, 1–4.6 mm ID and 1.7–10 μm particle size are employed in phenolic detection by advanced HPLC techniques like UHPLC (ultra-high pressure chromatography) and HTLC (high temperature liquid chromatography) and two-dimensional liquid chromatography (LC × LC) [212,256,257]. Most HPLC assays of phenolics are carried out at ambient column temperature. Recently, however, higher temperatures have also been recommended due to new columns and instrumentation [252,258]. HPLC running time is the other factor that influences the detection of phenolics and can range from 10 to 150 min. Roggero *et al.* [259] emphasized that high reproducibility of results when long analysis times are employed requires constant temperature. 

Phenolics are frequently identified using UV-VIS and photodiode array (PDA) detectors at wavelengths 190–380 nm [260,261] but fluorimetric (FLD) [262], colorometric arrays [263], PDA coupled with fluorescence [113] and chemical reaction detection techniques [264] are other methods used. Mass spectrometric (MS) detectors attached to high performance liquid chromatograph (HPLC–MS) [265,266], electrospray ionization mass spectrometry (ESI-MS) [217,267], matrix-assisted laser desorption/ionization mass spectrometry (MALDI–MS) [268,269], fast atom bombardment mass spectrometry (FAB-MS) [222,270] and electron impact mass spectrometry [270] have also been utilized for structural characterization and confirmation of different phenolic classes. HPLC coupled with MS detectors is highly sensitive and has the power to achieve high specificity due to the mass selectivity of detection [271]. HPLC–NMR and UHPLC are the other novel techniques to identify bioactive compounds in new sources of rare natural products [272,273,274]. The new trends in the analysis of phenolic compounds are hydrophilic interaction liquid chromatography (HILIC) as well as 2-dimensional liquid chromatography (2-D LC). HILIC may become more popular due to higher compatibility of applied mobile phase when linked to MS and enhanced accuracy to analyze polar components in complex matrices [275]. 2-D LC is a recent advance in chromatography that can afford separation and identification of structurally similar and minor compounds from complex matrices, enhancing peak capacity and selectivity [276]. A successful combination of 2-D LC × HILIC and 2-D LC × RP-LC has been used to detect polar and semi-polar fractions in traditional Chinese medicine. This combination of 2D-LC systems showed great potential to separate different components of a wide range of polarity from complex samples, which is not possible when using 1-D RPLC [277]. Table 9 presents some recent HPLC applications for determining different classes of phenolic compounds.

### 5.4. Other Assays for Separation and Quantification of Phenolics

Paper chromatography (PC) and thin-layer chromatography (TLC) are two partitioning techniques employed to separate phenolics in foods [182]. PC is a simple method and less utilized compared to HPLC and GC. PC has been used to separate and identify phenolic compounds from tea leaf with butanol/acetic acid/water as the mobile phase [278]. In other studies, flavonoids, phenolic acids and glycoflavones have been separated from three green leafy vegetables using PC [279].

TLC is a more powerful technique than PC to analyze phenolics, especially in crude plant extracts. Phenolics in crude plant extracts can be separated by a number of TLC techniques, which are cheap and provide for multiple detection on the same TLC plate in a short analysis time [120]. Sajewicz *et al.* [280] indicated that a silica gel TLC-based video imaging method is a valuable complementary fingerprint technique to identify phenolic acids and flavonoids fractions from different sage species. de Oliveira *et al.* [281] also utilized silica gel TLC to identify phenolic compounds from *Baccharis trimera* extract.

High-speed counter current chromatography (HSCCC) is a biphasic liquid-liquid partitioning method used to isolate and separate mixture components [28]. This unique technique has been widely used to purify and separate various natural compounds [282,283,284,285]. HSCCC separates compounds based on their partition coefficients between two solvent phases, which are determined by their hydrophobicity [286]. The method uses no solid support to allow permanent adsorption of sample compounds [287]. Furthermore, a crude extract can be applied to isolate and purify natural compounds without any preparation. Wang *et al.* [287] applied HSCCC to separate phenolics from a crude ethyl acetate extract of *Halimodendron halodendron* with chloroform-methanol-water-acetic acid (4:3:2:0.05, v/v) as the two-phase solvent system. Three phenolic compounds were successfully separated by HSCCC. He *et al.* [288] separated and purified four minor phenolic acids from black currant samples. A two-phase solvent system consisting of ethyl acetate/water/-*n*-hexane/methanol (15:7:5:4, v/v) gave an efficient separation. The chemical structures of the purified phenolic acids were confirmed by HPLC-MS and NMR.

Capillary electrophoresis (CE) is high-resolution technique conducted with a solution of ions in a narrow capillary column. It is suitable to identify charged low and medium-molecular-weight compounds rapidly and efficiently with high-resolution and has low sample and reagent volume requirements [289]. There are few studies on the use of CE to separate and identify phenolics in plant materials [290,291,292,293,294]. Micellar electrokinetic chromatography (MEKC), capillary electro chromatography (CEC) and capillary zone electrophoresis (CZE) coupled with ultraviolet detection (UV), and electrochemistry detection or mass spectrometry detection (MS) are the most widely used techniques among the different types of CE separation [295,296,297]. Yuan *et al.* [298] developed MEKC to determine the main lignans in different parts of *Schisandra sphenanthera.* CE-ESI-microTOF-MS has been optimized to separate and identify phenolic compounds in buckwheat [299].

The other versatile chromatographic technique used to analyze and identify phenolics is supercritical fluid chromatography (SFC). Compared to other well-known chromatographic techniques (HPLC and GC), SFC has high separation efficiency, high-resolution power, short analysis time, is environmentally friendly and is compatible with different types of detectors [300,301,302]. Eight polyphenols in grape seed extract have been identified using SFC by Kamangerpour *et al.* [303]. Liu *et al.* [304] also identified polyhydroxyl flavonoids and quercetin by packed-column SCF.

The utility of both GC and HPLC coupled with MS has been shown repeatedly for the detection of phenolic compounds in a range of samples [201,305]. HPLC and GC are very useful, accurate and suitable methods to determine phenolics in various kinds of foods because they give very reliable results within short analysis times. Moreover, with progress in the development of HPLC techniques, such as 2-D LC and HILIC, simultaneous identification of phenolics with a wide range of polarities will be possible. 

## 6. Conclusions

Cereal, vegetable and fruit consumption contributes to improved human health and lowers the risk of disease. These benefits may depend significantly on the phenolic content of these foods. The biology and health benefits of phenolics have lead researchers to discover, modify and utilize techniques for the extraction, separation and quantification of these compounds from natural sources. These methods need to be simple, rapid, environmentally friendly and comprehensive. This review has presented an overview of advanced extraction techniques to isolate and purify phenolics from plant-based sources, and provided information about some of the advanced separation and identification methods for plant phenolics. 
